# Supplementary material for: Combining ReACp53 with Carboplatin to Target High-Grade Serous Ovarian Cancers
Source: Cancers (Basel). 2021 Nov 24;13(23):5908. doi: 10.3390/cancers13235908 (PMC8657291; doi:10.3390/cancers13235908)
Supplement: Supplementary file 1 [file cancers-13-05908-s001.zip › Supplemental Figure 1.pdf]

Supplementary Figure 1

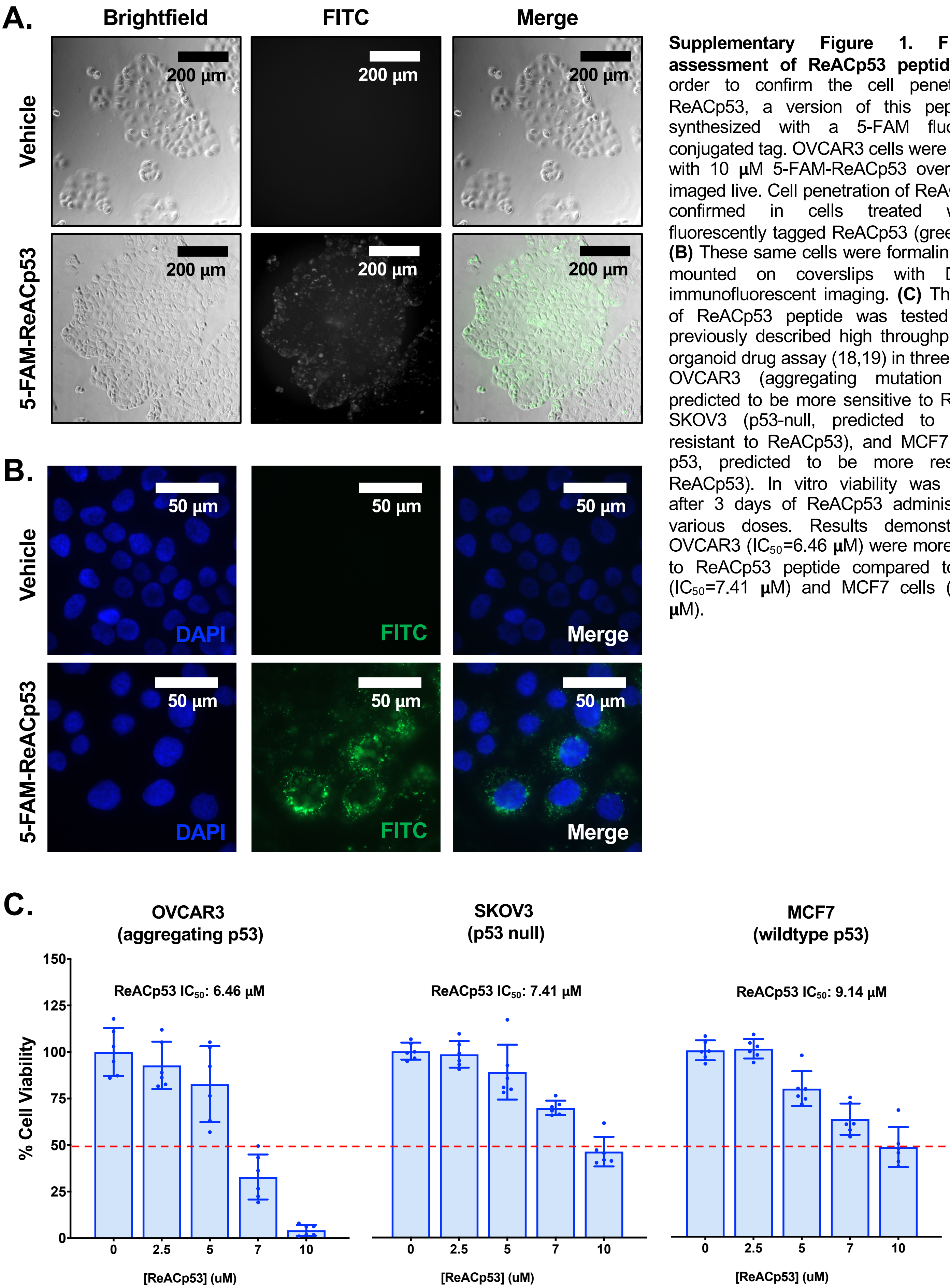

Supplementary Figure 1. Functional assessment of ReACp53 peptide. (A) In order to confirm the cell penetrance of ReACp53, a version of this peptide was synthesized with a 5-FAM fluorescence conjugated tag. OVCAR3 cells were incubated with 10 **µ**M 5-FAM-ReACp53 overnight and imaged live. Cell penetration of ReACp53 was confirmed in cells treated with the fluorescently tagged ReACp53 (green signal). **(B)** These same cells were formalin fixed and mounted on coverslips with DAPI for immunofluorescent imaging. **(C)** The efficacy of ReACp53 peptide was tested using a previously described high throughput in vitro organoid drug assay (18,19) in three cell lines: OVCAR3 (aggregating mutation in p53, predicted to be more sensitive to ReACp53), SKOV3 (p53-null, predicted to be more resistant to ReACp53), and MCF7 (wildtype p53, predicted to be more resistant to ReACp53). In vitro viability was assessed after 3 days of ReACp53 administration at various doses. Results demonstrate that OVCAR3 (IC_{50}=6.46 **µ**M) were more sensitive to ReACp53 peptide compared to SKOV3 (IC_{50}=7.41 **µ**M) and MCF7 cells (IC_{50}=9.14 **µ**M).
