# Supplementary material for: Combining ReACp53 with Carboplatin to Target High-Grade Serous Ovarian Cancers
Source: Cancers (Basel). 2021 Nov 24;13(23):5908. doi: 10.3390/cancers13235908 (PMC8657291; doi:10.3390/cancers13235908)
Supplement: Supplementary file 1 [file cancers-13-05908-s001.zip › Supplemental Figure 10.pdf]

Supplementary Figure 10

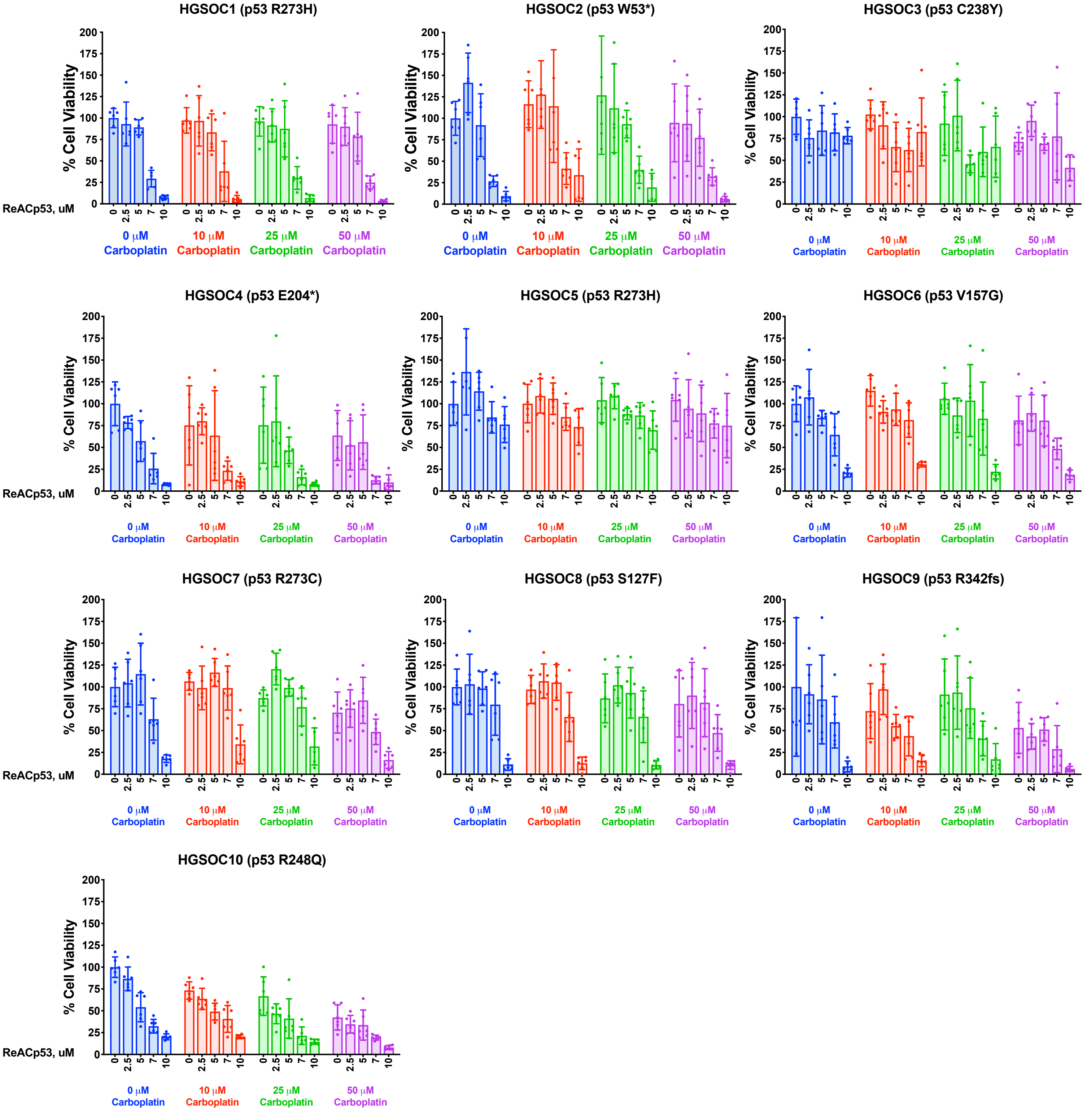

Supplementary Figure 10. Viability plots for human HGSOC tumors treated with the ReACp53 and carboplatin combination using the in vitro 3D miniring organoid drug assay. Individual cell viability data for all 10 primary patient HGSOC samples tested using the in vitro 3D miniring organoid drug assay. Data represents average measurements from 2 independent platings performed by separate investigators.
