# Supplementary material for: Combining ReACp53 with Carboplatin to Target High-Grade Serous Ovarian Cancers
Source: Cancers (Basel). 2021 Nov 24;13(23):5908. doi: 10.3390/cancers13235908 (PMC8657291; doi:10.3390/cancers13235908)
Supplement: Supplementary file 1 [file cancers-13-05908-s001.zip › Supplemental Figure 11.pdf]

Supplementary Figure 11

A.

Cell Line	p53 Mutation	p53 Aggregation Status
**Kuramochi**	**D281Y**	Unknown
**OVCAR8**	**Y126_K132del**	Unknown
**OVCAR4**	**L130V**	Unknown
**OVCAR3**	**R248Q**	Aggregating†
**OAW28**	**P152fs**	Unknown
**SKOV3**	**P89fs**	Unknown
**SNU-119**	**P151A**	Unknown
**CaOV3**	**Q136***	Unknown

† Xu J, Reumers J, Couceiro JR, De Smet F, Gallardo R, Rudyak S, et al. Gain of function of mutant p53 by coaggregation with multiple tumor suppressors. Nat Chem Biol. 2011 May;7(5):285–95.

B.

Patient Tumor Sample	p53 Mutation	p53 Aggregation Status
**HGSOC1**	**R273H**	Aggregating‡
**HGSOC2**	**W53***	Unknown
**HGSOC3**	**C238Y**	Unknown
**HGSOC4**	**E204***	Unknown
**HGSOC5**	**R273H**	Aggregating‡
**HGSOC6**	**V157G**	Unknown
**HGSOC7**	**R273C**	Suggested§
**HGSOC8**	**S127F**	Unknown
**HGSOC9**	**R342fs**	Unknown
**HGSOC10**	**R248Q**	Aggregating†

† Xu J, Reumers J, Couceiro JR, De Smet F, Gallardo R, Rudyak S, et al. Gain of function of mutant p53 by coaggregation with multiple tumor suppressors. Nat Chem Biol. 2011 May;7(5):285–95.

‡ Levy CB, Stumbo AC, Ano Bom AP, Portari EA, Cordeiro Y, Silva JL, et al. Co-localization of mutant p53 and amyloid-like protein aggregates in breast tumors. Int J Biochem Cell Biol. 2011 Jan;43(1):60–4.

§ Palanikumar L, Karpauskaite L, Al-Sayegh M, Chehade I, Alam M, Hassan S, et al. Protein mimetic amyloid inhibitor potently abrogates cancer-associated mutant p53 aggregation and restores tumor suppressor function. Nat Commun. 2021 Jun 25;12(1):3962.

Supplementary Figure 11. TP53 mutations and predicted aggregation statuses of all ovarian cancer cell lines and patient HGSOC specimens tested using the in vitro organoid drug assay. The p53 mutation status and aggregation potential for **(A)** ovarian cancer cell lines and **(B)** primary patient HGSOC tumor samples tested using the in vitro organoid drug assay. TP53 mutation statuses for cell lines were obtained from the IARC TP53 database. Patient TP53 mutations were determined by clinical sequencing or by whole exome sequencing. The aggregating status for some TP53 mutations has previously been suggested or demonstrated experimentally other investigators. The aggregation potential for vast majority of mutations in p53 are unknown.
