# Supplementary material for: Combining ReACp53 with Carboplatin to Target High-Grade Serous Ovarian Cancers
Source: Cancers (Basel). 2021 Nov 24;13(23):5908. doi: 10.3390/cancers13235908 (PMC8657291; doi:10.3390/cancers13235908)
Supplement: Supplementary file 1 [file cancers-13-05908-s001.zip › Supplemental Figure 2.pdf]

Supplementary Figure 2

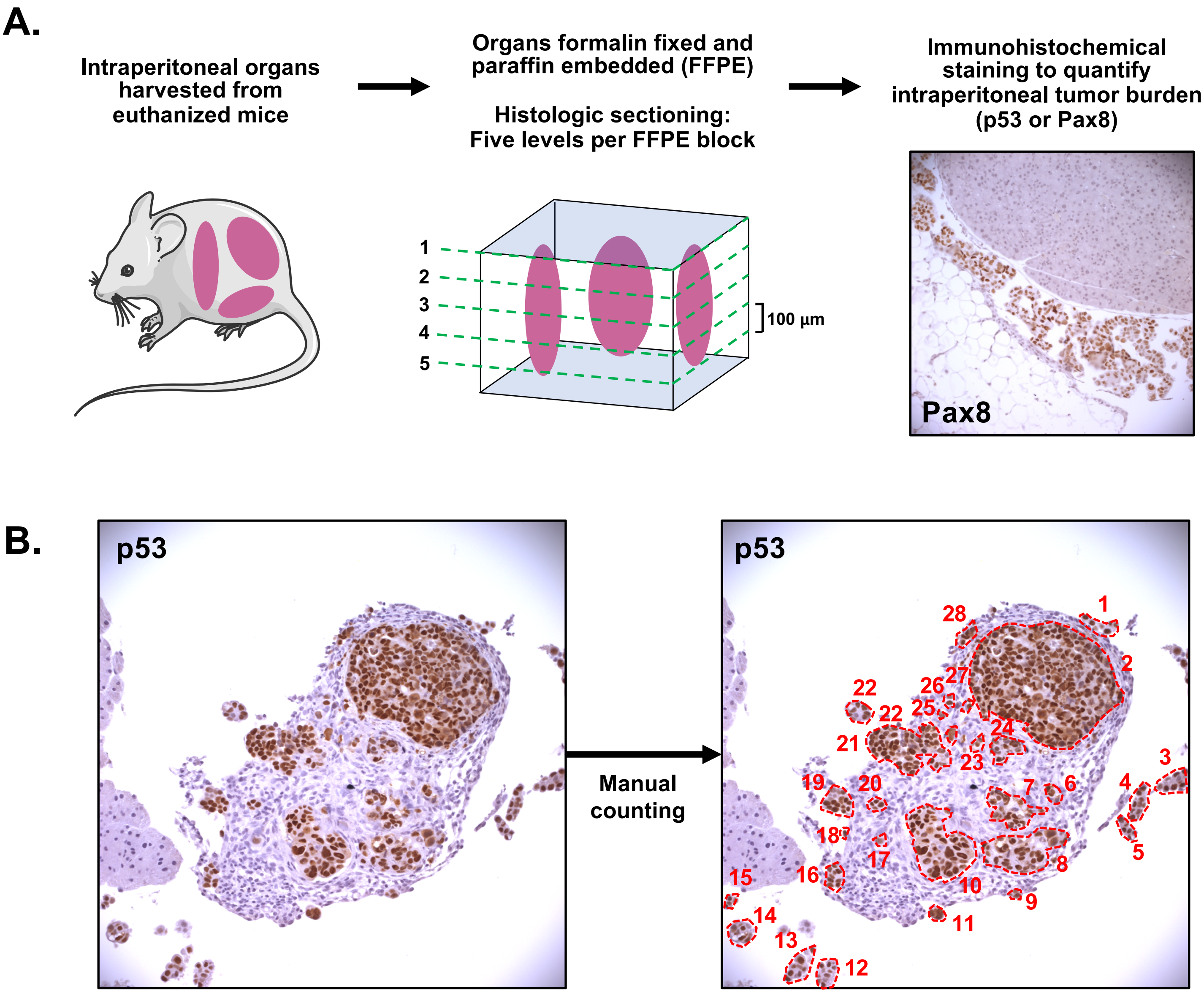

Supplementary Figure 2. Quantification of tumor burden using histologic intraperitoneal organ sections from euthanized mice. (A) Schema for quantification of intraperitoneal metastases. Portions of intraperitoneal organs (ovary, uterus, fat pad, spleen, pancreas, liver, and peritoneum) were dissected from euthanized mice, formalin fixed and paraffin embedded (FFPE). FFPE blocks containing organs were histologically sectioned at 5 levels 100 µm apart throughout the depth of the block. Histologic slides were stained for p53 or Pax8 expression using immunohistochemistry. Example image shown for Pax8 staining of OVCAR3 tumor implants. **(B)** IHC-stained slides were individually inspected and tumor implants were counted manually across the whole slide by two independent investigators. Individual implants were defined as continuous regions of positive staining. Non-continuous positive regions that were near one another were counted as separate implants. Example of one field of view shown for p53-stained organs.
