# Supplementary material for: Combining ReACp53 with Carboplatin to Target High-Grade Serous Ovarian Cancers
Source: Cancers (Basel). 2021 Nov 24;13(23):5908. doi: 10.3390/cancers13235908 (PMC8657291; doi:10.3390/cancers13235908)

Supplementary Figure 3

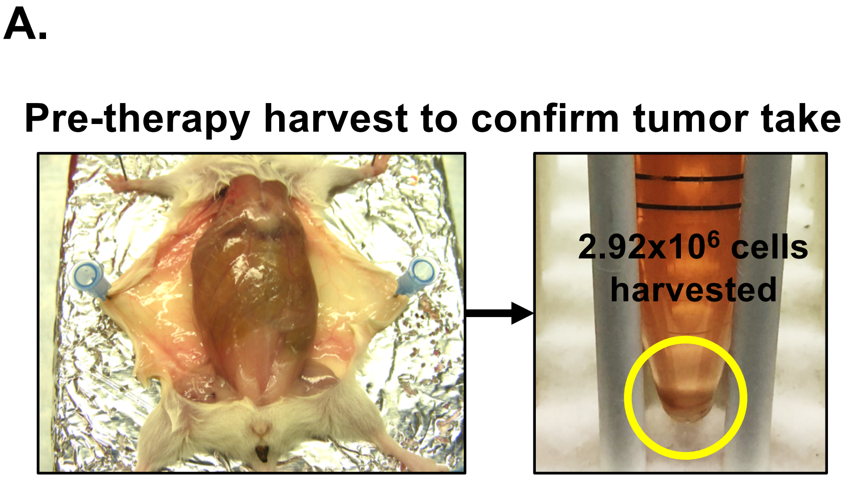

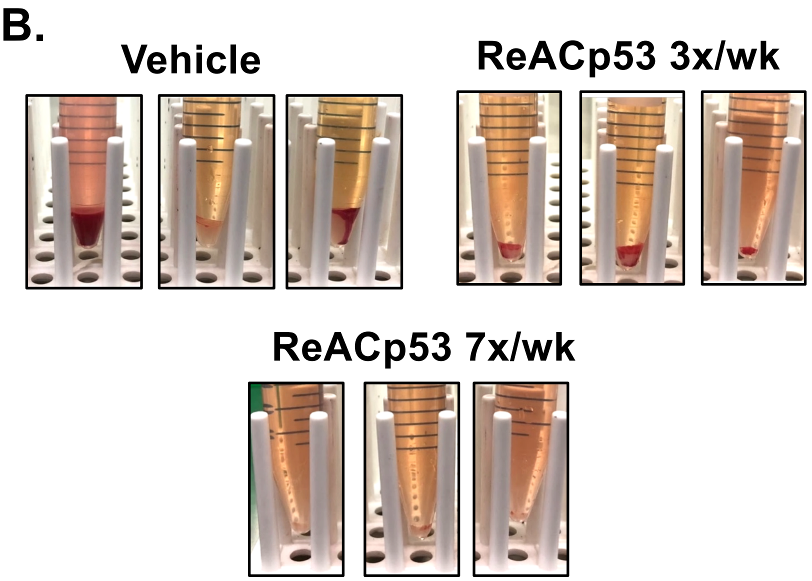

Supplementary Figure 3. Pelvic wash, Trop1+ cell pellet quantification, and histologic analysis of organs harvested from treated mice. (A) n=13 mice were injected with 1.0×10^6 OVCAR3 tumor cells. After two weeks, one mouse was randomly selected and harvested to confirm tumor take. **(B)** Remaining OVCAR3 tumor-bearing mice were randomized to receive either vehicle, ReACp53 3x/week, or ReACp53 7x/week (n=4 per treatment). After 3 weeks of treatment, residual tumors were harvested. Example of cells harvested from pelvic washings from one animal per treatment group is shown in Figure 1. Other cell pellets for euthanized mice are shown. **(C)** Cells retrieved from pelvic washings were immunostained for Trop1 and analyzed by flow cytometry. The percentage of Trop1+ cells for each treatment group is shown, indicating the majority of retrieved cells are epithelial tumor cells. **(D)** Organs were histologically examined and tumor was confirmed by p53 staining using immunohistochemistry (red arrows). Representative images for tumor organ implants shown for each treatment group.

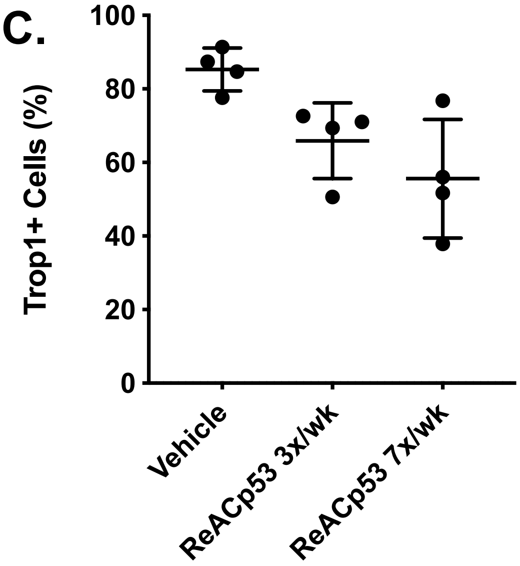

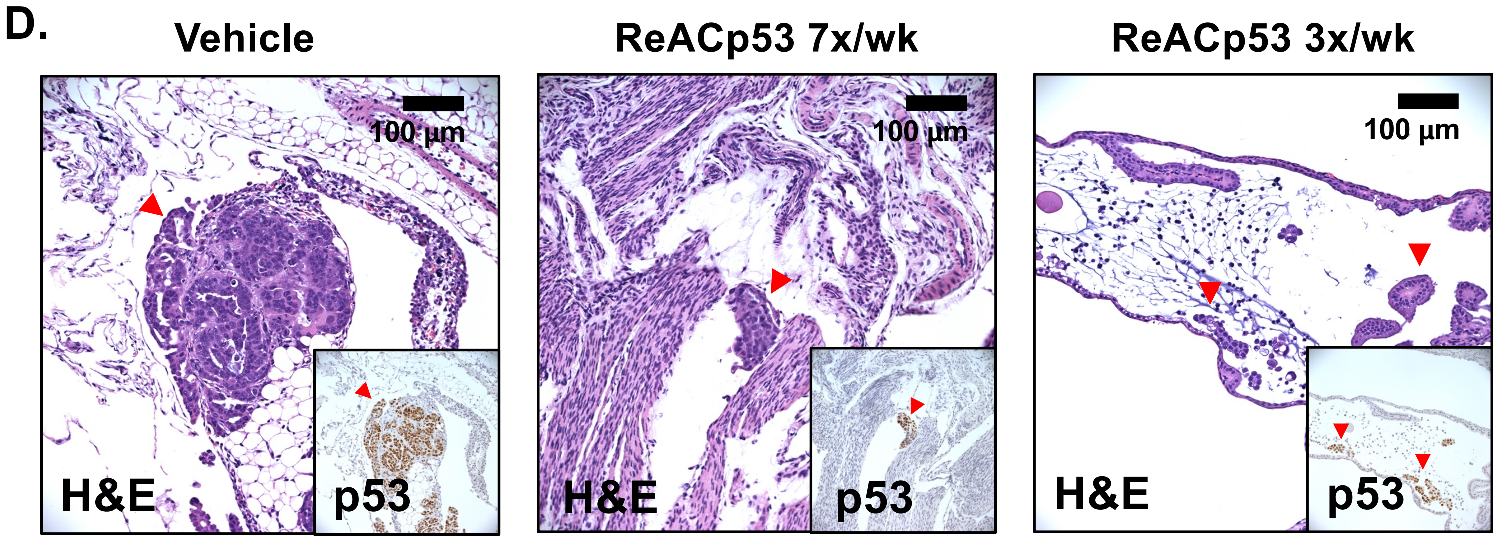

Supplement: Supplementary file 1 [file cancers-13-05908-s001.zip › Supplemental Figure 3.pdf]
