# Supplementary material for: Combining ReACp53 with Carboplatin to Target High-Grade Serous Ovarian Cancers
Source: Cancers (Basel). 2021 Nov 24;13(23):5908. doi: 10.3390/cancers13235908 (PMC8657291; doi:10.3390/cancers13235908)
Supplement: Supplementary file 1 [file cancers-13-05908-s001.zip › Supplemental Figure 4.pdf]

Supplementary Figure 4

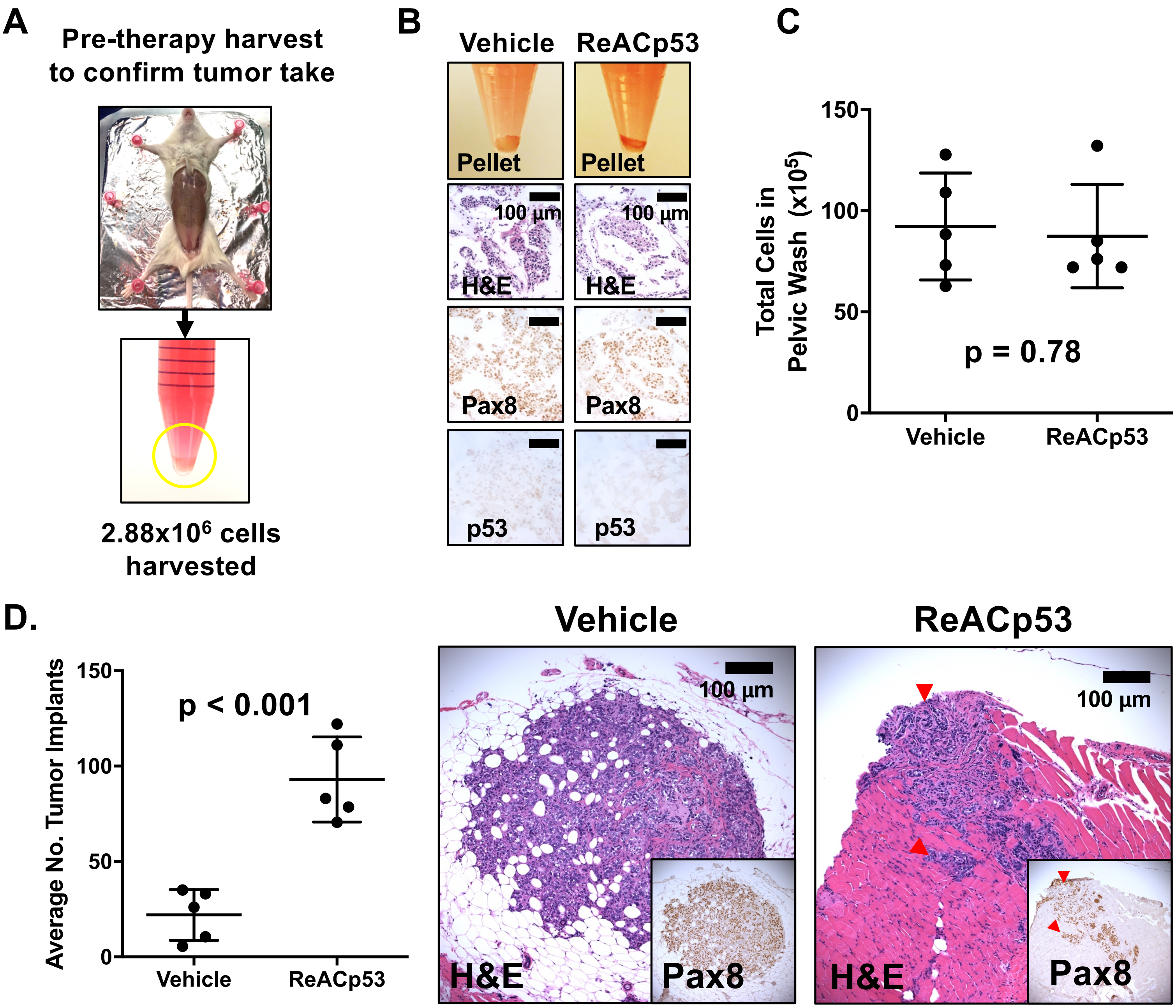

Supplementary Figure 4. ReACp53 did not target SKOV3 tumors in vivo. Xenografts were established by injecting 1.0x10^6 SKOV3 cells (p53-null) into the intraperitoneal (IP) space of n=11 NSG mice. **(A)** Two weeks after tumor establishment, one mouse was euthanized to confirm tumor take. The remaining n=10 mice/cell line were randomized to receive either vehicle or ReACp53 15 mg/kg 3x/week IP for four weeks (n=5/treatment). At the end of therapy, mice were euthanized, IP tumors were harvested by peritoneal lavage, and organs were formalin-fixed and paraffin embedded (FFPE) for histologic analysis. **(B)** Representative cell pellets. Immunostaining for Pax8 confirms the presence of tumor cells. SKOV3 cells are p53-null. **(C)** Quantification of peritoneal lavage demonstrated equivalent numbers of cells in mice treated with ReACp53 vs. vehicle (p=0.78). **(D)** The average number of tumor implants was higher in mice treated with ReACp53 vs. vehicle (p<0.001). Pax8 immunostaining was performed on organs from euthanized mice to assess tumor burden (red arrows).
