# Supplementary material for: Combining ReACp53 with Carboplatin to Target High-Grade Serous Ovarian Cancers
Source: Cancers (Basel). 2021 Nov 24;13(23):5908. doi: 10.3390/cancers13235908 (PMC8657291; doi:10.3390/cancers13235908)
Supplement: Supplementary file 1 [file cancers-13-05908-s001.zip › Supplemental Figure 5.pdf]

Supplementary Figure 5

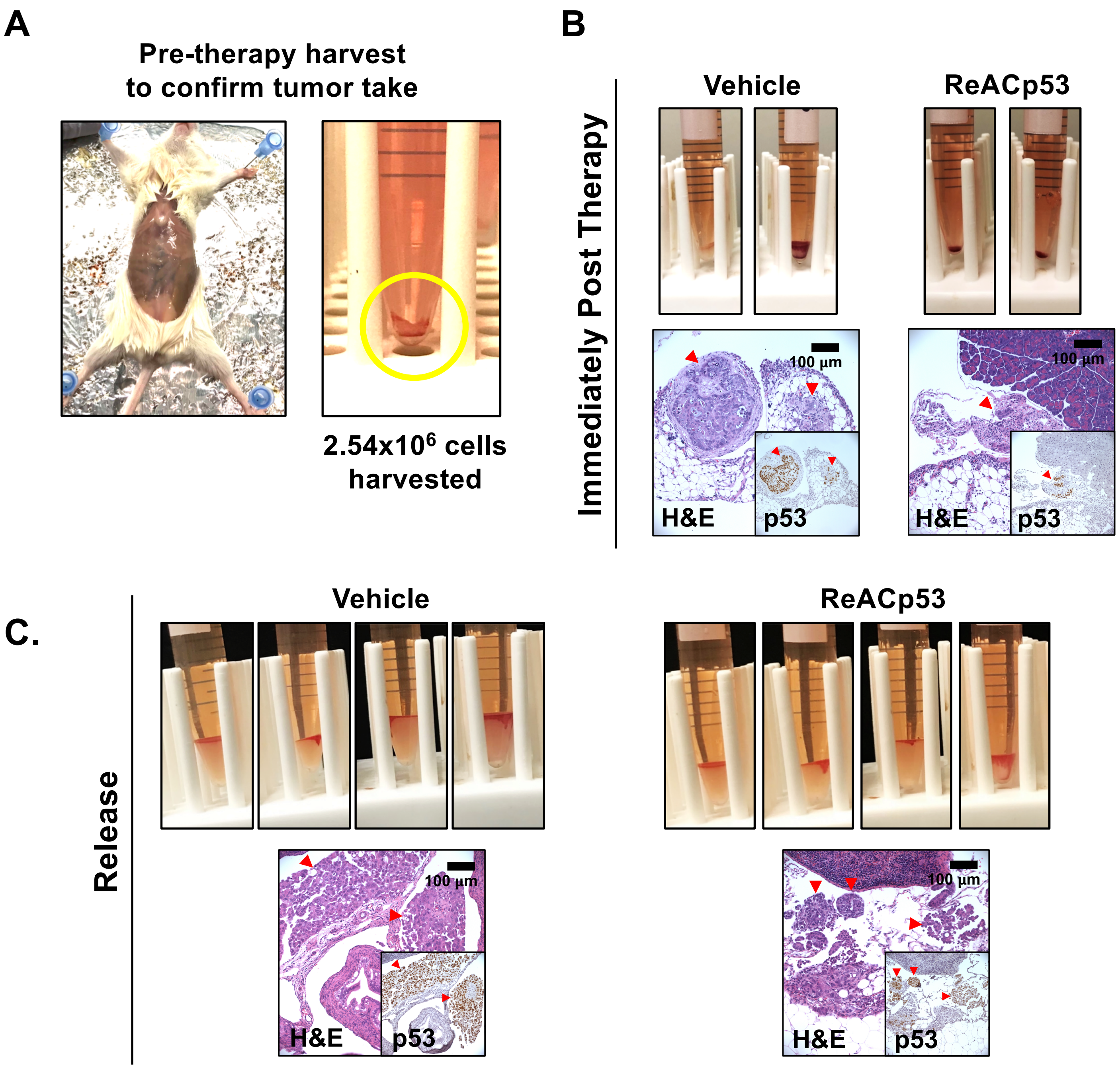

Supplementary Figure 5. Histologic analysis of organs harvested from mice bearing OVCAR3 tumors immediately post therapy and after four weeks off-therapy. (A) n= 17 Mice were injected with 1.0x10^6 OVCAR3 tumor cells. After two weeks, one mouse was randomly selected and euthanized pre-therapy to confirm tumor take. **(B)** The remaining mice bearing OVCAR3 xenografts were randomized to receive either vehicle or ReACp53 15mg/kg 3x/week IP (n=8 per treatment). After 4 weeks of therapy, residual tumors were harvested from n=3 mice/treatment (immediately post therapy cohort). Intraperitoneal (IP) cells were collected by pelvic wash. Representative pelvic washing cell pellet shown for one mouse per treatment in Figure 2, remaining n=2 mice/treatment harvested shown. Representative images for metastatic organ implants (red arrows) shown for each treatment group. **(C)** The remaining n=5 mice/treatment were released off-therapy for an additional 4 weeks (release cohort). IP tumors were collected and analyzed by pelvic wash. Representative images of pelvic washing shown in Figure 2, remaining cell pellets n=4 mice/treatment shown. Representative images for metastatic organ implants (red arrows) shown for each treatment group.
