# Supplementary material for: Combining ReACp53 with Carboplatin to Target High-Grade Serous Ovarian Cancers
Source: Cancers (Basel). 2021 Nov 24;13(23):5908. doi: 10.3390/cancers13235908 (PMC8657291; doi:10.3390/cancers13235908)

Supplementary Figure 6

A.

Cell Line	Platinum Sensitivity
**OVCAR3**	**Sensitive**
**OVCAR4**	**Intermediate**
**OAW28**	**Resistant**
**Kuramochi**	**Resistant**
**OVCAR8**	**Resistant**
**SKOV3**	**Intermediate**
**SNU-119**	**Resistant**
**CaOV3**	**Intermediate**

Data summarized from:
Beaufort et al. PLoS ONE. 2014 Sep 17
Haley et al. Oncotarget. 2016 Apr 27;7(22):32810–20.

Supplementary Figure 6. Viability plots for human ovarian cancer cell lines tested in the in vitro 3D miniring organoid drug assay. (A) Platinum sensitivity for each cell line was extracted from literature as indicated (24, 25). **(B)** Cell viability data for all ovarian cancer cell lines tested using the in vitro 3D miniring organoid drug assay. Data shown represent an average of 5 independent platings by 2 separate investigators.

B.

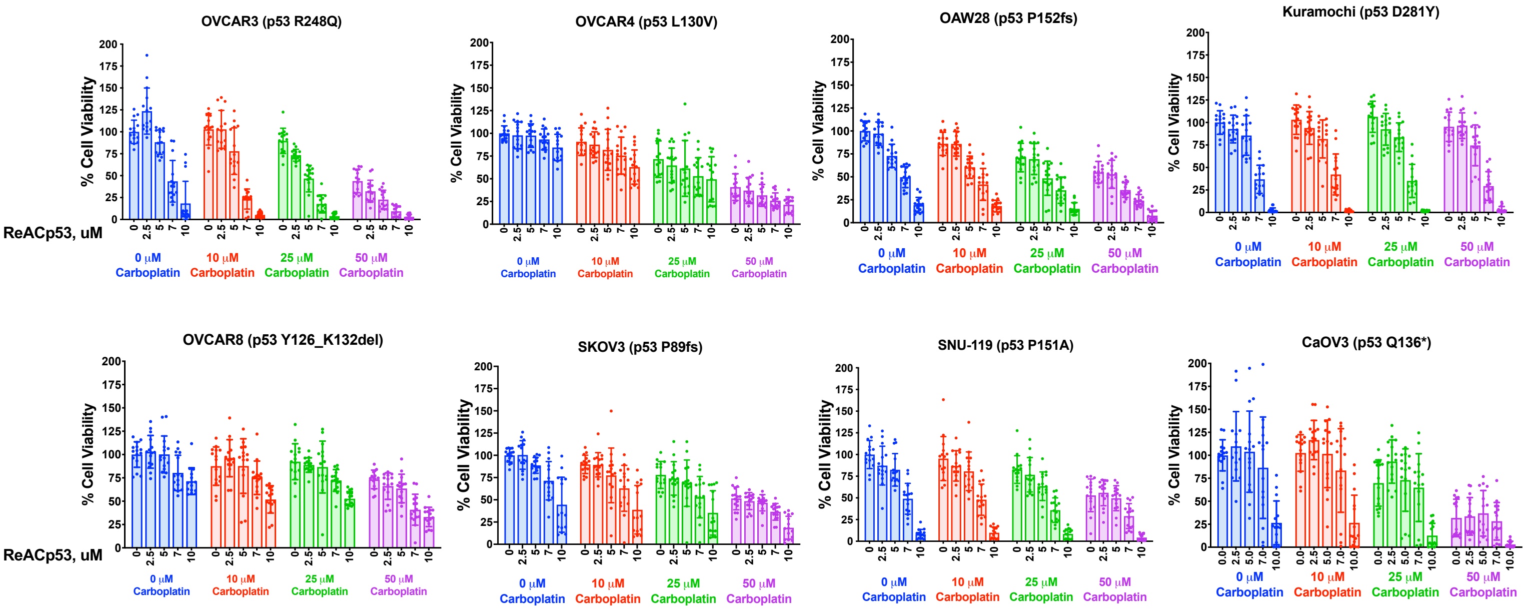

Supplement: Supplementary file 1 [file cancers-13-05908-s001.zip › Supplemental Figure 6.pdf]
