# Supplementary material for: Combining ReACp53 with Carboplatin to Target High-Grade Serous Ovarian Cancers
Source: Cancers (Basel). 2021 Nov 24;13(23):5908. doi: 10.3390/cancers13235908 (PMC8657291; doi:10.3390/cancers13235908)
Supplement: Supplementary file 1 [file cancers-13-05908-s001.zip › Supplemental Figure 7.pdf]

Supplementary Figure 7

A.

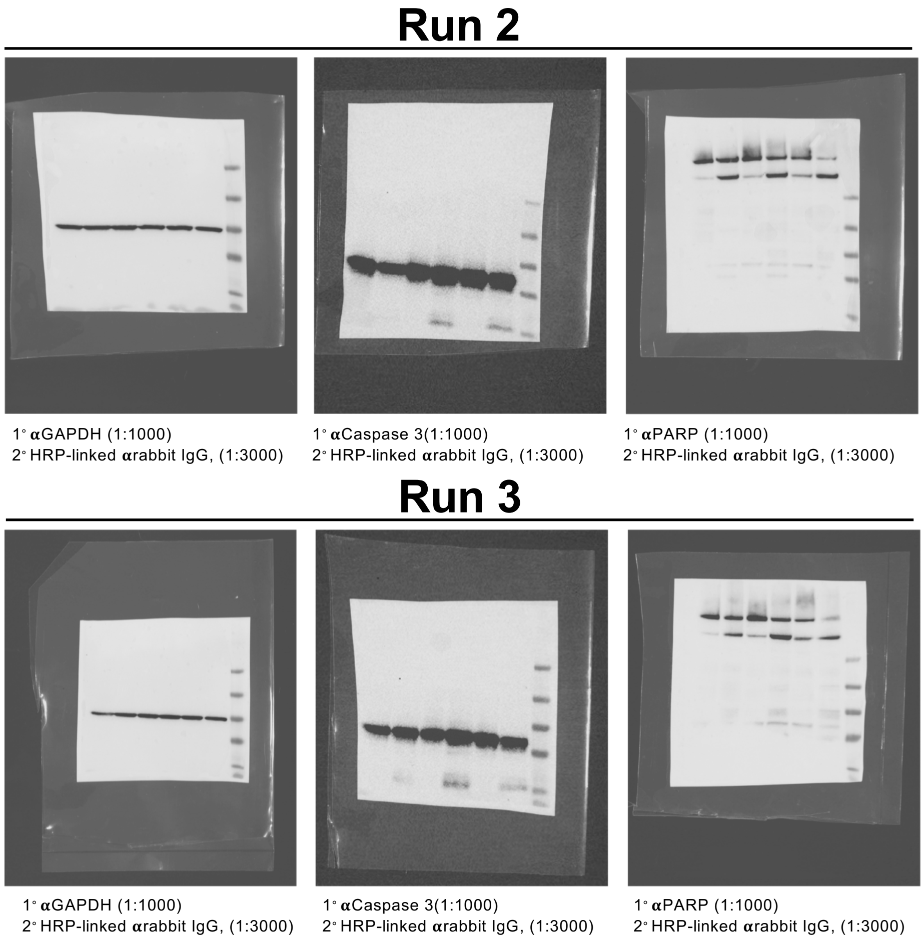

C.

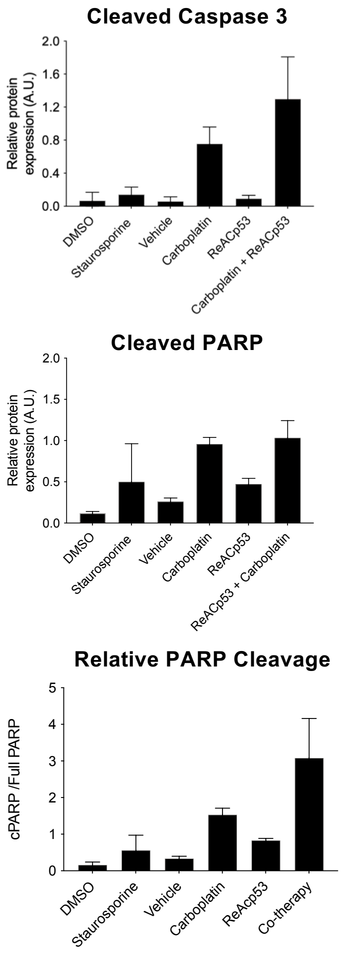

B.

Lane	Loading
1	DMSO
2	1 uM Staurosporine
3	Vehicle
4	50 uM Carboplatin
5	4 uM ReACp53
6	4uM ReACp53 + 50 uM Carboplatin
7	Ladder

Supplementary Figure 7. Western blots for detecting apoptosis markers in treated OVCAR3 organoids. OVCAR3 organoids embedded in matrigel were treated with vehicle, ReACp53 (4uM), carboplatin (50uM), or the combination of the two for 72 hours with daily drug replenishment. Other organoids were treated with staurosporine or its control, DMSO. Following treatment, organoids were released from matrigel using dispase and lysed in RIPA buffer and protease inhibitors. Lysate was fractionated on polyacrylamide gel, and PARP, caspase 3, and GAPDH were detected by western blot using antibodies as indicated. **(A)** Full western blots for two independent experiments. Data from run 1 is already shown in Figure 3E. Protein loading as indicated in **(B). (C)** Quantification of cleaved caspase 3, cleaved PARP, and relative PARP cleavage defined as the ratio of cleaved-PARP divided by full length PARP, normalized to GAPDH as loading control - average data from 3 runs. No statistical significance was found using the non-parametric Kruskal-Wallis test.
