# Supplementary material for: Combining ReACp53 with Carboplatin to Target High-Grade Serous Ovarian Cancers
Source: Cancers (Basel). 2021 Nov 24;13(23):5908. doi: 10.3390/cancers13235908 (PMC8657291; doi:10.3390/cancers13235908)
Supplement: Supplementary file 1 [file cancers-13-05908-s001.zip › Supplemental Figure 8.pdf]

Supplementary Figure 8

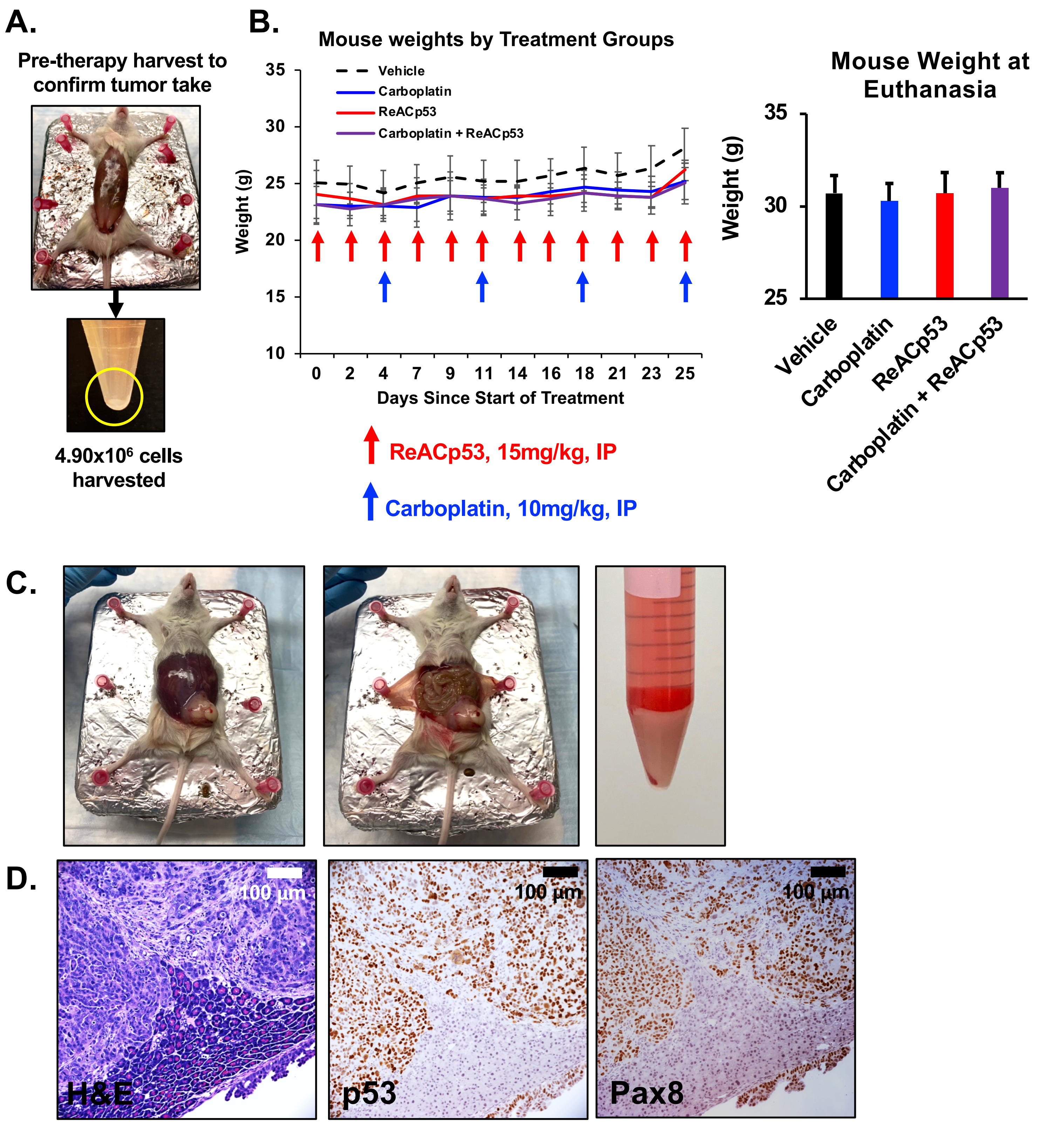

Supplementary Figure 8. Survival analysis of mice bearing OVCAR3 intraperitoneal xenografts. 29 NSG mice were injected with 3.0x10^6 OVCAR3 tumor cells. **(A)** After two weeks of tumor establishment n=1 mouse was randomly selected and euthanized to confirm tumor take. **(B)** Mice were treated with either vehicle, ReACp53, carboplatin, or the combination for 4 weeks and then released off-therapy and euthanized upon reaching NIH-defined endpoint criteria. Mouse weights measured throughout the treatment phase demonstrate no substantial weight loss (line graph). Mouse weights were again recorded at the time of euthanasia (bar graph). **(C)** Representative images of mouse necropsy. Upon reaching endpoint, mice presented with large ascites burden (often bloody) and numerous gross tumor implants. **(D)** Representative images of tumor organ implants. Immunohistochemical staining of Pax8 and p53 positivity confirm presence of tumor cells.
