# Supplementary material for: Combining ReACp53 with Carboplatin to Target High-Grade Serous Ovarian Cancers
Source: Cancers (Basel). 2021 Nov 24;13(23):5908. doi: 10.3390/cancers13235908 (PMC8657291; doi:10.3390/cancers13235908)
Supplement: Supplementary file 1 [file cancers-13-05908-s001.zip › Supplemental Figure 9.pdf]

Supplementary Figure 9

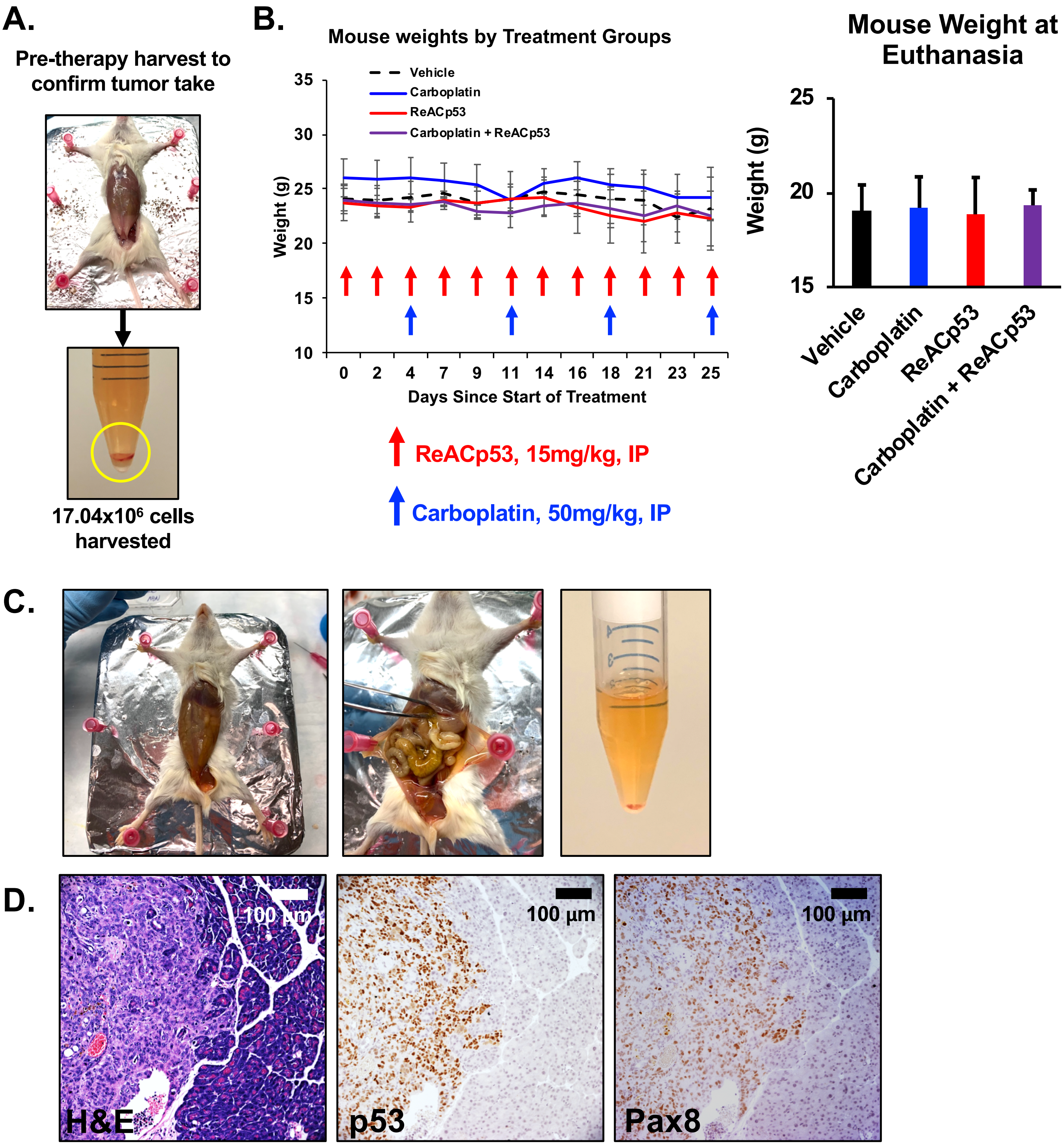

Supplementary Figure 9. Survival of OVCAR8-bearing mice. Mice were injected with 3.0×10^6 OVCAR8 tumor cells (n=29) **(A)** One mouse was randomly selected and euthanized to confirm tumor take. **(B)** Remaining mice randomized to receive a four week course of either vehicle, ReACp53, carboplatin, or the ReACp53 and carboplatin combination (n=7 mice/treatment). Following treatment, therapy was stopped and mice were euthanized based on NIH-defined endpoint criteria. Mouse weights measured throughout treatment phase demonstrate an overall downward trend (line graph). Mice were weighted at endpoint (bar graph). **(C)** Representative images of mouse necropsy. Mice presented with a jaundiced appearance, low ascites burden, presence of intraperitoneal bilirubin, and notable gross tumor implants in some cases. **(D)** Representative images of tumor organ implants. Pax8 and p53 immunostaining confirm presence of tumor cells.
